# Viral diarrhea in Ghana: a scoping review of disease burden, epidemiology, and control strategies

**DOI:** 10.3389/fpubh.2026.1840821

**Published:** 2026-07-29

**Authors:** Alberta Afua Kekeli Ayebi, Sabastine Eugene Arthur, Michelle Yeboah-Manu, Monica Baaba Jones, George Boateng Kyei

**Affiliations:** 1 Medical and Scientific Research Center, University of Ghana Medical Center, Accra, Ghana; 2 College of Health Sciences, University of Ghana, Accra, Ghana; 3 Department of Virology, Noguchi Memorial Institute for Medical Research, University of Ghana, Accra, Ghana; 4 Departments of Medicine and Molecular Microbiology, Washington University School of Medicine, St. Louis, MO, United States

**Keywords:** adenovirus, astrovirus, Ghana, norovirus, rotavirus, viral diarrhea

## Abstract

Viral diarrhea remains a significant public health concern in Ghana, particularly among children under 5 years of age. While rotavirus remains the leading cause, the growing impact of other viral pathogens, such as norovirus, adenovirus, and astrovirus, necessitates expanded research and intervention efforts. The introduction of the rotavirus vaccine has significantly reduced diarrhea prevalence, but persistent disparities in disease burden highlight the need for comprehensive control strategies. This scoping review maps the existing evidence on the burden, evolution, and control measures of viral diarrhea in Ghana. A systematic search of PubMed, ScienceDirect, and Scopus was conducted to identify relevant studies published between 1996 and 2025. Findings from local studies indicate that, despite improvements in sanitation, access to potable water, and vaccination programs, viral diarrhea remains widespread and unevenly distributed across populations. The review underscores the importance of enhanced diagnostic capacity, robust surveillance systems, and targeted prevention strategies to effectively combat this public health challenge. By synthesizing available evidence, this scoping review highlights knowledge gaps and priority areas for future research, policy, and intervention in Ghana's fight against viral diarrhea.

## 1 Introduction

Diarrhoeal diseases remain a significant public health challenge worldwide, particularly in low-and middle-income countries (LMICs), where they contribute substantially to childhood morbidity and mortality. According to the World Health Organization (WHO), diarrhea is defined as the passage of at least three loose stools per day or an increase in stool frequency beyond an individual's typical pattern (1). Despite global efforts to improve sanitation, access to clean water, and healthcare infrastructure, diarrhoeal diseases still account for an estimated 1.7 billion cases annually in children, resulting in nearly half a million deaths (1).

In Ghana, diarrhea remains one of the leading causes of outpatient visits and childhood hospitalizations, with viral pathogens playing a significant role in disease burden. Among the various aetiological agents of infectious diarrhea, viruses such as rotavirus, norovirus, astrovirus, and adenovirus account for a substantial proportion of cases, particularly in children under 5 years of age (2, 3). While interventions such as the introduction of the rotavirus vaccine have significantly reduced diarrhea-related morbidity and mortality, other viral pathogens continue to pose a growing threat, underscoring the need for enhanced surveillance and diagnostic capacity.

Despite progress in controlling diarrhoeal diseases through vaccination and improved healthcare interventions, Ghana still faces several challenges, including limited epidemiological data, diagnostic constraints, and disparities in healthcare access. These gaps hinder effective disease management and the development of targeted interventions.

This review aims to provide a comprehensive assessment of viral diarrhoeal diseases in Ghana, focusing on their epidemiology, diagnostic approaches, and the impact of current interventions. By identifying research gaps and highlighting areas for improvement, this study seeks to inform public health strategies to reduce the burden of viral diarrhea in the country.

## 2 Methodology

### 2.1 Review framework

This scoping review was conducted following the methodological framework proposed by Arksey and O'Malley methodology (4) and reported according to PRISMA-ScR guidelines.

### 2.2 Search strategy

**Figure 1:**
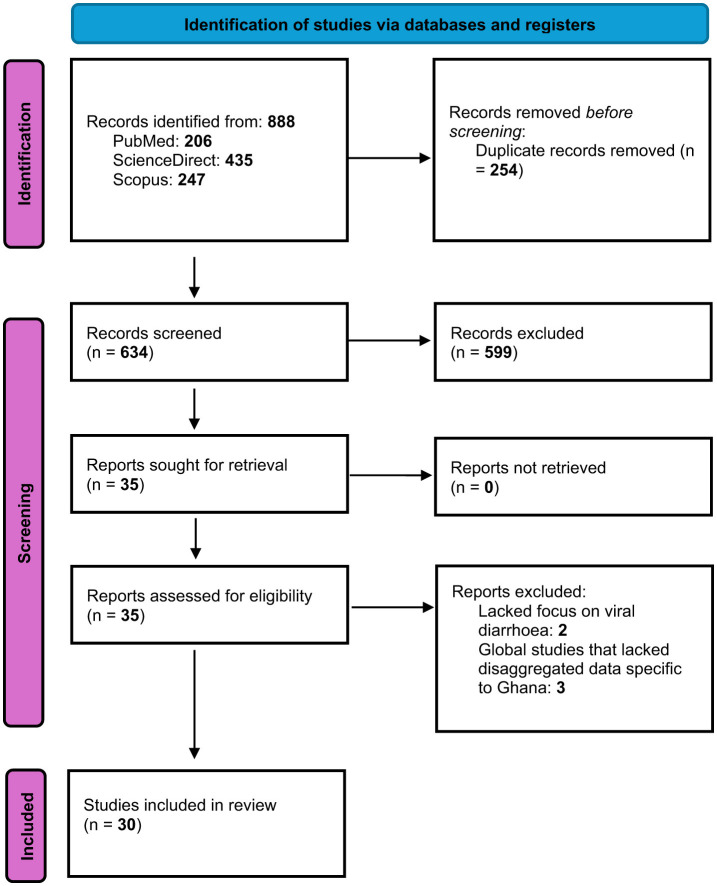
PRISMA flow chart.

A comprehensive search was conducted across PubMed, ScienceDirect, and Scopus, between 31st March to 7th April 2025 to identify relevant studies published between January 1996 and March 2025, providing sufficient breadth to examine historical and contemporary trends in viral diarrhea in Ghana while maintaining a manageable scope for synthesis. While only studies within this timeframe were included in the evidence mapping, we also cited a small number of materials published after March 2025 in the discussion section to provide additional context. These later publications were not part of the formal scoping review dataset. Search strings combined controlled vocabulary and keywords related to diarrhea, viral pathogens (rotavirus, norovirus, adenovirus, astrovirus, sapovirus), epidemiology, burden, prevalence, incidence, surveillance, and Ghana. The full search strings are provided in the supplementary materials. To ensure relevance and methodological rigor, the search was restricted to peer-reviewed research articles and review papers. Editorials, commentaries, conference abstracts, and other non-research publications were excluded. Reference lists of included studies were also screened to identify additional eligible publications. The search yielded 888 records (PubMed: 206; ScienceDirect: 435; Scopus 247). After removal of 254 duplicates, 634 records were screened by title and abstract. The PRISMA 2020 flow diagram (attached) summarizes the selection process (Figure 1).

### 2.3 Inclusion criteria

Studies were included if they reported:

1. Incidence, prevalence, or distribution of viral diarrhea in Ghana.

2. Specific viral pathogens (rotavirus, norovirus, adenovirus, astrovirus, sapovirus).

3. Impact of rotavirus vaccination on the burden of viral diarrhea.

4. Rotavirus serotypes before and after vaccine introduction.

### 2.4 Exclusion criteria

Articles were excluded if they:

1. Focused on non-viral causes of diarrhea.

2. Reported only global or pooled estimates without disaggregated data specific to Ghana.

3. Did not provide primary data or relevant outcomes.

### 2.5 Study selection

All records identified through the search were imported into Rayyan for screening and data management. Screening was conducted independently by two reviewers at both the title/ abstract and full-text stages. Any discrepancies were discussed, and when consensus could not be reached, a third reviewer served as arbiter. The review protocol was not prospectively registered, and no a priori protocol was deposited in a public repository. Although protocol registration is encouraged to promote transparency, the exploratory nature of this scoping review led the authors to proceed without formal registration. In line with PRISMA-ScR guidance, no formal quality appraisal was undertaken, as the objective was to map the breadth of available evidence rather than assess study validity. Of the 35 reports assessed for eligibility, 30 studies met the inclusion criteria, while 5 were excluded (2 lacked focus on viral diarrhea; 3 were global studies without Ghana-specific data).

### 2.6 Data extraction

Data extraction was carried out using a standardized form developed for this review. From each included study, information was charted on study characteristics such as author, year of publication, design, and setting, as well as details of the study population including age group and sample size. Variables relating to the type of viral diarrhea investigated, incidence and prevalence estimates, and distribution patterns were recorded. Attention was given to data on the impact of rotavirus vaccination and the distribution of rotavirus genotypes before and after vaccine introduction. Key findings and conclusions from each study were also documented. To ensure accuracy and consistency, data extraction was performed by one reviewer and independently verified by a second.

The selected papers were subsequently categorized into the following thematic areas: the prevalence of viral diarrhoeal pathogens; the impact of rotavirus vaccination, assessing both changes in infection rates and genotype variation; and the available diagnostic techniques for establishing the etiology of viral diarrhea.

This structured approach facilitated a comprehensive synthesis of the evidence on the burden and control of viral diarrhea in Ghana.

## 3 Results

### 3.1 Burden of viral diarrhea in Ghana

#### 3.1.1 Prevalence and incidence of viral diarrhea in children under 5 years of age

The true prevalence of viral diarrhea in Ghana remains unknown as the etiology of diarrhea is not routinely investigated at health facilities in Ghana. Despite this, diarrhoeal diseases are a major public health concern in Ghana, contributing significantly to morbidity and mortality, particularly among children under 5 years of age. The Ghana Demographic and Health Survey estimated that the prevalence of diarrhea among children under 5 years of age was 13% (5, 6). The number, however, is thought to be a severe underestimation, and as such, independent studies usually show higher figures (7). A surveillance study conducted by Akuffo et al., from 2010 to 2012 at the 37 Military Hospital and Tamale Teaching Hospital, reported that 84.3% of diarrhoeal cases were associated with viral pathogens, including rotavirus, norovirus, adenovirus, and astrovirus (3). A study by Bandoh et al., estimated that the prevalence of diarrhea among children under 5 years of age in 5 coastal cities in Accra was 36% (8). Yet again, another study investigating the prevalence of diarrhea across Ghana estimates the prevalence of diarrhoeal cases to range from 4 to 7304 per 10,000 people (9). Despite uncertainty about the true prevalence of viral diarrhea in children, available studies suggest that viral pathogens contribute substantially to childhood diarrhoeal disease in Ghana (3, 10, 11). One such study estimated that rotavirus accounted for about 58% of reported diarrhoeal cases at a health facility (12). Yet another study evaluating diarrhea cases requiring hospitalization over 13 months at the Korle Bu Teaching Hospital found that Rotavirus accounted for 34% of such cases (13). Using data from these studies, it can be inferred that the prevalence of viral diarrhea in Ghana is also high.

#### 3.1.2 Morbidity and mortality of viral diarrhea in children under 5 years of age

Viral diarrhea has a significant effect on the morbidity and mortality rate in children under 5 years of age in Ghana. A review of the District Health Information Management System (DHIMS) revealed that a total of 5,645,533 diarrhoeal cases were reported between February 2018 and March 2022 (14). Another study conducted at the Korle Bu Teaching Hospital (KBTH) and the Komfo Anokye Teaching Hospital (KATH) found that rotavirus accounted for about 48% of admissible diarrhoeal cases in the hospitals (10). Similarly, another study conducted at the Princess Marie Louise Children's Hospital, La General Hospital, and Mamprobi Polyclinic found that 58% of diarrhoeal cases in their outpatient departments were due to rotavirus infection (12). Despite its relatively high morbidity rate, diarrhoeal diseases in general have a relatively lower mortality rate. Regardless, diarrhoeal diseases remain a top 10 cause of mortality in children under 5 years of age in Ghana (15, 16). There is not much information on the mortality rate of viral diarrhea nationally; however, studies have provided estimates of rotavirus mortality in the country. Globally, rotavirus is estimated to be responsible for 40% of diarrhea-related deaths, with most occurring in lower-middle-income countries like Ghana (17). In 2021, diarrhoeal deaths were estimated to be highest in South Asia, followed by sub-Saharan Africa, with rotavirus being the most common causative pathogen across all age groups (18). In Ghana, a study assessing rotavirus gastroenteritis requiring admission in Korle Bu Teaching Hospital estimated the case fatality at 2.9% (13). Additionally, a study evaluating global, regional, and national rotavirus mortality between 2000 and 2013 estimated the rotavirus diarrhea mortality rate in Ghana to be 10–50 deaths per 100,000 children (17).

### 3.2 Distribution of viral diarrhea in Ghana

A study using data from the 2014 Ghana Demographic Health Survey found that diarrhoeal diseases in Ghana were most prevalent among male children aged 12–23 months living in rural areas (19). Additionally, maternal factors such as younger maternal age (< 20 years) and completion of secondary school education were associated with an increased risk of childhood diarrhea (19). The authors attributed the rural-urban disparity in diarrhea prevalence to inadequate access to safe water, poor sanitation, and poor hygiene practices (19).

Although diarrhoeal diseases generally peak in the dry season, additional peaks have been observed during the rainy months, particularly in June and October. These seasonal variations may be linked to factors such as flooding and overcrowding in affected areas, which contribute to poor environmental and personal hygiene, ultimately leading to diarrhea outbreaks.

Regionally, studies have reported a higher incidence of diarrhoeal diseases in northern Ghana. This may be attributed to widespread open defecation, high rates of childhood malnutrition, elevated poverty levels, limited infrastructural development, and inadequate coverage of water, sanitation, and hygiene (WASH) programs (11, 20, 21). Despite these spatial and temporal variations in diarrhea prevalence, the predominant causative organisms remain relatively stable.

### 3.3 Etiology of viral diarrhea in Ghana

Rotavirus is the most common viral cause of diarrhea in Ghana, affecting both rural and urban settings, with additional contributions from noroviruses, adenoviruses, and astroviruses (11, 22). In our search, most studies on viral diarrhea have focused on rotavirus. A few aimed to determine all viruses present in diarrhoeal cases, and about two studies focused specifically on norovirus.

The prevalence of other viral diarrhoeal agents is shown in Table 1. Of key importance is norovirus, whose prevalence increased significantly post initiation of rotavirus vaccination, from an upper limit of 15.9% to 48.3%. The GII genotype was approximately 77%-82% in these studies, with GII.4 being the most prevalent subtype. Adenovirus prevalence ranged from 3.5% to 28%, with higher prevalence in older studies that used RT-PCR methods, compared with the relatively new studies that used only ELISA or Rapid Antigen Tests. Adenovirus groups D and F were most prevalent, with subtypes AdV F40 and F41 being the most common for the latter group. The prevalence of astrovirus was assessed in relatively fewer studies and was less than 10%. AstV-8 made up almost 50%, followed by AstV-2 with about 33% and AstV-5 with 17%.

**Table 1:** Summary of the major viral causes of diarrhea, their prevalence, transmission mode, and clinical features.

Viral pathogen	Prevalence in Ghana (%)	Transmission mode	Key symptoms	Reference
Rotavirus	Refer to Table 3	Fecal-oral, contaminated food/water	Severe watery diarrhea, vomiting, fever, dehydration	(10, 23)
Norovirus	6.8%−15.9% (pre-vaccine) → 48.3% (post-vaccine)	Fecal-oral, foodborne, direct contact	Vomiting, diarrhea, nausea, abdominal cramps	(3, 22, 27, 91–93)
Adenovirus	3.5%−28%	Fecal-oral, respiratory droplets	Prolonged diarrhea, fever, dehydration	(3, 22, 27, 42, 94)
Astrovirus	0.4%–%7.5%	Fecal-oral, contaminated food/water	Mild diarrhea, vomiting, fever	(3, 22, 27, 42)

Monitoring studies conducted at two referral hospitals in the northern and southern regions of Ghana reported that viral diarrhea accounted for approximately 85% of hospitalized diarrhea cases, with rotavirus alone contributing nearly 60% (3, 22). Similarly, another study in the northern region estimated the prevalence of rotavirus in diarrhoeal cases at 55% (22). In Ghana, rotavirus infection accounts for 32%−50% of diarrhea-related hospital admissions, with the highest prevalence in the northern region (49%) and the lowest in the Volta region (10%) (10, 23).

Seasonally, rotavirus infections peak between December and February, with a secondary peak from May to July (24–26). Prior to the introduction of vaccination, common rotavirus genotypes circulating in Ghana included G1, G2, G3, G9, and P[4], P[6], P[8], P[9], with the G1P[8] genotype combination being the most prevalent (13, 23, 27, 28). A few studies detected G10 and G12 genotypes pre-vaccination but with a relatively lesser prevalence (23, 29).

### 3.4 Diagnosis of viral diarrhoeal diseases in Ghana

In Ghana, as in many parts of the world, diarrhoeal diseases predominantly affect children, with a significant proportion of cases being viral in origin and often self-limiting. Hence, most of these cases do not end up in health facilities (30, 31). Effective management of diarrhea requires an accurate diagnosis through clinical history and examination, as well as appropriate clinical intervention (31, 32). However, in most Ghanaian healthcare settings, pathogen-specific diagnostic testing is not routinely performed. Instead, diarrhea management is largely based on clinical symptoms, with primary interventions focused on rehydration.

#### 3.4.1 Current diagnostic practices

Unlike the developed countries, where laboratory-based pathogen identification is standard practice, most hospitals in Ghana do not conduct routine diagnostic testing for diarrhoeal pathogens. Specific pathogen testing is typically available only in select clinical settings or within the framework of research and surveillance studies. This diagnostic gap limits the ability to accurately determine the etiology of diarrhea, which is crucial for targeted treatment and epidemiological surveillance. Table 2 summarizes the diagnostic tests typically used in Ghana and the settings in which they are used. Diagnostic methods were standardized into three categories: molecular methods (PCR, RT-PCR, qPCR, semi-nested PCR), immunologic methods (EIA, ELISA, antigen assays), and microscopy-based methods.

**Table 2:** Comparison of available diagnostic techniques, their advantages, and limitations.

Diagnostic method	Pathogens detected	Advantages	Limitations	Availability in Ghana	Reference
Immunologic method (rapid antigen tests)	Rotavirus, adenovirus	Quick results (15–30 min), low cost	Low sensitivity for norovirus and astrovirus	Limited to research and select hospitals	(12, 23)
Molecular methods (PCR, RT-PCR, qPCR, semi-nested PCR)	All viral diarrhea pathogens	High sensitivity and specificity	Expensive, requires specialized labs	Available in research settings	(95, 96)
Immunologic methods (EIA, ELISA)	Rotavirus, norovirus, adenovirus	Good sensitivity, useful for surveillance	Requires trained personnel	Limited to major hospitals	(10, 92)
Stool microscopy	None (only detects parasites, not viruses)	Inexpensive, widely available	Cannot detect viral pathogens	Available in most health facilities	(22, 93)

PCR, polymerase chain reaction; RT-PCR, reverse transcription polymerase chain reaction; qPCR, quantitative polymerase chain reaction; ELISA, enzyme-linked immunosorbent assay; EIA, enzyme immunoassay.

#### 3.4.2 National and international guidelines on diagnosis

Ghana adopted the WHO/UNICEF guidelines for the management of acute diarrhea in children in 2010. These guidelines emphasize clinical diagnosis, severity assessment, and management strategies but do not recommend routine pathogen testing (33). Similarly, Ghana's Standard Treatment Guidelines (STG, 2017) provide diagnostic clues to help determine the etiology of acute diarrhea but do not require pathogen-specific testing for diagnosis and management (34).

#### 3.4.3 Challenges and implications of limited diagnostic testing

The lack of widespread laboratory-based pathogen testing presents significant challenges. Without routine testing, distinguishing between viral and bacterial diarrhea is difficult, which may lead to unnecessary antibiotic use and hinder accurate epidemiological surveillance of viral diarrhea. Expanding access to rapid diagnostic tools, such as PCR, ELISA, or antigen-based tests, could improve case identification and guide public health interventions.

### 3.5 Public health interventions against viral diarrhea

#### 3.5.1 Rotavirus vaccination in Ghana

As stated previously, rotavirus is a leading cause of severe diarrhea, contributing significantly to morbidity and mortality among children worldwide. The introduction of rotavirus vaccines has markedly reduced infant mortality by decreasing both the incidence and severity of rotavirus infections. The first rotavirus vaccine, RotaShield, was licensed globally in 1998 but was withdrawn a year later due to adverse events. In 2006, two new vaccines, Rotarix (GlaxoSmithKline) and RotaTeq (Merck), were introduced following extensive clinical trials. Although these vaccines showed lower efficacy in Africa and Asia, the World Health Organization (WHO) recommended their use in 2009, citing their substantial public health benefits. In 2018, two additional vaccines, Rotavac (Bharat Biotech) and Rotasiil (Serum Institute), both developed in India, were recommended for use in low-income countries (35).

#### 3.5.2 Implementation of rotavirus vaccination in Ghana

Ghana introduced the rotavirus vaccine into its national immunization program in 2012, initially adopting the two-dose Rotarix schedule targeting G1P[8] and non-G1 genotypes. In 2020, Ghana transitioned to the three-dose Rotavac schedule, which is comparable in efficacy and safety to Rotarix but offers a cost advantage. The uptake of Rotavac has been similar to that of Rotarix, and the incidence of diarrhea has remained relatively stable following the switch (36).

#### 3.5.3 Impact of rotavirus vaccination on diarrhoeal disease burden

Since the introduction of the rotavirus vaccine in 2012, Ghana has observed a significant decrease in diarrhoeal diseases among children under 5 years of age. According to the Ghana Demographic Health Survey, the incidence of diarrhea in this age group declined from 20% in 2008 to 12% in 2014. Rotavirus vaccination coverage among children aged 12–23 months increased from 88.5% in 2014 to 93% in 2022 (5, 37, 38).

An interrupted time-series analysis by Armah et al. compared rotavirus-related hospital admissions across seven sentinel sites in Ghana, revealing a reduction from 48% to 28% among children under 5 years of age (10). Notably, while the prevalence of diarrhea is highest among children aged 12–23 months, rotavirus prevalence is highest among those under 1 year (10).

Similarly, a study monitoring trends in two major pediatric hospitals in southern Ghana reported a decrease in rotavirus-related admissions from nearly 50% to 28%. The vaccine had the greatest impact on children under 18 months (24). A multisite study conducted in 7 hospitals across the northern, middle, and southern belts of Ghana recorded the highest rotavirus prevalence, 60.4% before vaccination and 39.6% post-vaccination (39). Another study assessed rotavirus prevalence pre- and post-vaccination in two teaching hospitals and found it to be about 50% and 29%, respectively (40). Studies evaluating the prevalence of rotavirus infection before or after the introduction of the rotavirus vaccine were more numerous. The prevalence before vaccination ranged from 16.2% in the Ashanti Region to 58% in the Greater Accra Region. Post-vaccination, prevalence recorded in studies has ranged from 14.5% to 39.0%. Studies conducted in more recent years seem to record lower prevalence. Table 3. Summarizes the prevalence studies on rotavirus before and after the introduction of the vaccine.

**Table 3:** Effect of rotavirus vaccination on rotavirus diarrhea across regions.

Study region	Year of study	Pre-rotavirus vaccination prevalence	Post-rotavirus vaccination prevalence	Reference
Multisite	2009–2016	60.4%	39.6%	(39)
Multisite (KBTH; KATH)	2009–2016	50%	29%	(40)
Greater Accra (KBTH, PML)	2008–2014	49.7%	27.8%	(24)
	1992	15.1%	Not assessed	(97)
	2004–2005	34%	Not assessed	(13)
	2006–2011	48.9%	Not assessed	(29)
	2007–2011	48.2%	Not assessed	(23)
	2008–2009	49.2	Not assessed	(11)
	2009	58%	Not assessed	(12)
	2013–2014	Not assessed	31%	(98)
	2014–2016	Not assessed	39%	(43)
	2017–2018	Not assessed	22.5%	(99)
	2017–2018	Not assessed	32.1%	(100)
	2019	Not assessed	23.7%	(42)
Ashanti	2007–2008	16.6%	Not assessed	(93)
	2012–2014	Not assessed	26.2%	(94)
	2014–2015	Not assessed	11%	(101)
Northern	1997–2002	40.5%	Not assessed	(102)
	1998–2000	39%	Not assessed	(103)
	2003–2004	39.8%	Not assessed	(28)
	2005–2006	55%	Not assessed	(22)
	2005–2006	38.7%	Not assessed	(27)
	2020–2021	Not assessed	14.8%	(25)
Multisite (Accra, Kumasi, Ho, Navrongo)	2009–2014	48%	28%	(10)
Multisite (Accra, Tamale)	2010–2012	27.9%	Not assessed	(3)

KBTH, korle-bu teaching hospital; KATH, komfo anokye teaching hospital; PML, princess marie louie hospital. Pre-rotavirus vaccination period, before may 2012; Post-rotavirus vaccination period, after may 2012.

#### 3.5.4 Regional variations in vaccine impact

The impact of rotavirus vaccination has varied across different regions in Ghana. Asare et. al, used mathematical models to assess the impact of rotavirus vaccination in Ghana. In Accra, the reduction in rotavirus infections has ranged from 55% to 71%, while in Kumasi, the decrease has been between 36% and 55%. Conversely, the Northern region, particularly Navrongo, has experienced a more modest reduction of 12% to 20% in severe rotavirus gastroenteritis cases over the past decade (41). Several factors may contribute to these regional disparities, including earlier exposure to natural rotavirus infection, shorter duration of maternal immunity, and lower vaccine coverage (41).

Among studies evaluating rotavirus prevalence after the introduction of the vaccine in the Greater Accra Region, the most recent survey, conducted in 2019, reported a low prevalence of 14.5% (42). However, one study reported a prevalence of 39%, which is almost as high as the pre-vaccination prevalence observed in some studies. This result was likely influenced by the study site, Ashaiman, which is known to be overpopulated with poor social amenities. Also, the vaccination status of the children used in the study was not reported (43). One multisite study conducted in the post-vaccination era across facilities in the Greater Accra, Western, and Northern regions recorded an overall prevalence of 27.7%; however, the prevalence in the Western region was 50.8%, 36% in the Northern region, and 21.6% in the Greater Accra region (44). These show the dissimilarities in the impact of rotavirus vaccination in Ghana.

#### 3.5.5 Impact of rotavirus vaccination on rotavirus genotypes

The prevalent rotavirus genotypes and genotype combinations in Ghana before and after the introduction of the rotavirus vaccine are shown in Tables 4, 5, respectively. The most prevalent G-genotype found in most studies was G1, followed closely by G2 and G3. For the P-genotype, the commonest were P[6] and P[8]. The prevalent genotype combinations were G1P[8], G2P[6] and G3P[6]. Post introduction of rotavirus vaccination, G-genotypes G12 and G10 are gaining ascendancy. There has not been much change in the P-genotypes. The G12P[8], G10P[6], and G3P[6] genotype combinations are increasing in the post-vaccination era.

**Table 4:** Rotavirus genotypes prior rotavirus vaccination.

Year of study and reference	Dominant G-genotypes	Dominant P-genotypes	Dominant G and P combination types
1997–2000. (104)	G2–28.9% G9–25.4% G3–16.6% G1–8.1%	P[8]−40.9% P[6]−33.8% P[4]−13.8%	G9P[8]−22.6% G2P[6]−21.1% G3P[4]−12.4% G2P[8]−5.6%
1998. (105)	G3–78% G2–14% G1–2%	P[4]−72.3% P[6]−21.3% P[8]−2.1%	G3P[4]−74% G2P[6]−12% G3P[6]−7%
1999. (106)	G2–54.3%	P[6]−82.6%	G1P6–50.0%
2003–2004. (28)	G1- 42% G2–15.9% G3–15.9%	P[6]−36.2% P[8]−27.5% P[9]−10.1%	G1P[8]−17.4% G3P[6]−11.6% G1P[6]−11.6%
2004–2005. (13)	G2–22.9% G1−17.1% G9–17.1% G3–12.9%	P[6]−38.6% P[8]−21.4% P[4]−4.3%	G9P[6]−10% G1P[8]−8.6%
2005–2006. (27)	G1–90.1% G12–3.5%	P[8]−85.9% P[6]−5.6%	GIP[8]−78.9% G1P[6]−5.6%
2006–2011. (29)	G1–50.9% G2–18.8%	P[8]−36.1% P[6]−30.7%	G1P[8]−36.1% G3P[6]−9% G2P[6]−7%
2007–2011. (23)	G1–43% G2–15.9% G3–10.8%	P[8]−29.2% P[6]−24.9% P[4]−7.2%	G1P8–20.2% G3P6–6.4% G2P6–5.1%
2008–2009. (11)	G1–41.7% G2–19.5% G3–8.8%	P[6]−34.5% P[8]−19.8% P[4]−9.4%	G1P[8]−14.8%
2009. (12)	G3–25%	P[6]−18.8%	G3P[6]−18.8% G2P[6]−12.5%
2009-2016. (39)	G1–40.1% G3–13.9% G2–12.5%	P[8]−34.6% P[6]−30.7% P[4]−5.0%	G1P[8]−20.0% G3P[6]−9.2%

**Table 5:** Rotavirus genotypes after rotavirus vaccine introduction.

Year of study and reference	Dominant G-genotypes	Dominant P-genotypes	Dominant G and P combination types
2009–2016. (39)	G12–32.6% G1–16.3% G10–12.8%	P[8]−42.6% P[6]−33.0% P[4]−14.8%	G12P[8]−25.6% G10P[6]−10.0%
2014–2016. (43)	G3–39% G12–15% G1–14% G9–10%	P[6]−51% P[8]−28% P[4]−9%	G3P[6]−35% G12P[8]−14% G1P[8]−10% G9P[4]−9%
2019. (42)	G2–40% G1–33.5% G12–26.7%	P[4]−66.7% P[8]−22.2%	G2P[4]−67%
2020–2021. (25)	G10–41.3% G12–18% G3–15.4% G1–10.3% G4–5.1%	P[6]−62.9% P[8]−31.4% P[9]−5.7%	G10P[6]−41% G12P[8]−18% G3P[6]−16% G1P[8]−10% G4P[9]−5%

#### 3.5.6 Comparison with other countries

In 2009, the WHO recommended the use of rotavirus vaccine globally, and since then about 43 African countries have implemented the rotavirus vaccine in their immunization schedules (45). Since then, several African countries have documented a reduction in rotavirus diarrheal deaths and hospitalizations within 2–3 years post-rotavirus vaccine introduction (46, 47). A 7-year hospital surveillance in two Kenyan hospitals found a 57% reduction in hospitalization due to rotavirus diarrhea at Kilifi and a 59% reduction at Siaya. This trend continued the next year with Kilifi and Siaya recording a 80% and 82% reduction respectively (46). In Zimbabwe, after rotavirus vaccination was implemented in 2014, the percentage of rotavirus-positive visits declined by 40% and 43% among children 0–11 months of age and by 21% and 33% among children 12–23 months of age in 2015 and 2016, respectively (48). The Gambia introduced rotavirus vaccination in 2013. Before this, the prevalence of rotavirus in hospital admissions was 22%, which decreased to 11% post-vaccination. In severe hospitalized cases, the prevalence dropped from 33% to 8% following vaccine introduction, with the most pronounced effect observed in children under 1 year of age (49). The vaccine was also introduced in 2014 in Burkina Faso and since then hospitalizations that tested positive for rotavirus declined significantly among children < 5 years of age, from 36% in 2014 to 22% in 2015 and 20% in 2016 (50). Similarly in Senegal, sentinel surveillance recorded a 76% reduction in rotavirus positive hospitalizations between 2015–2016 and a 59% reduction between 2016-2017 following introduction of the vaccine in its nation immunization program (51). The findings support observations reported in Ghana regarding the beneficial impact of rotavirus vaccination.

#### 3.5.7 Other public health interventions

In view of the many devastating effects of diarrhoeal diseases, several public health interventions have been introduced to help reduce their incidence. Apart from rotavirus vaccination, which specifically targets viral diarrhea, these interventions target diarrhoeal diseases in general. They have, however, still had a significant impact on the burden and management of viral gastroenteritis in the country.

The WHO estimates that about 88% of diarrhoeal-related deaths can be attributed to unsafe water, inadequate sanitation, and poor hygiene (52). Unfortunately, other statistics have shown that 1.5 billion people still do not have access to basic sanitation services, with 419 million of them participating in open defecation (1). Additionally, in 2020, approximately 44% of the household wastewater produced globally was discharged without safe treatment, and at least 10% of the global population is thought to have consumed food irrigated with untreated sewage (1). In view of this, one of the most common and effective policies has been the introduction of Water, Sanitation and Hygiene (WASH) interventions (53). These include providing potable water, improved toilet facilities, and proper waste management to at-risk communities. WASH interventions remain popular, particularly as several studies have demonstrated that they reduce diarrhea prevalence in communities by 15% to 26% across all age groups (54). An example of such an initiative in Ghana is the Handwashing with Ananse game, developed by the Engagement Lab at Emerson College in collaboration with UNICEF Ghana, the Red Cross/Red Crescent Climate Center, and the Ghana Education Service (GES) to educate children on hand hygiene (55). This initiative is estimated to have caused an 8.2% reduction in self-reported illness including diarrhea in children who took part in the game as compared to other children who did not (56). Another example of WASH initiatives that have been implemented in Ghana is the Community-Led Total Sanitation (CLTS) which was introduced in Ghana in 2007 by Plan Ghana, the Community Water and Sanitation Agency (CWSA), and the Ministry of Local Government and Rural Development (MLGRD). Though the policy did not have significant effect on national figures, this policy was found to be effective in small remote villages with 61% of these villages achieving an Open Defecation Free status. Additionally, communities which successfully maintained their toilet facilities also saw a significant drop in diarrheal diseases in children (54). Another study in Ghana found an 11% reduction in the national incidence of diarrhoeal disease during the COVID- 19 pandemic, when strict hand hygiene protocols were implemented (14).

Breastfeeding has also been proven to be a cost-effective way to reduce diarrhea in children under 5 years of age. A study involving children from 9 sub-Saharan African countries, including Nigeria, found that early initiation of breastfeeding and exclusive breastfeeding were associated with a decreased prevalence of diarrhea (57). In contrast, children who had been introduced to complementary foods and children who continued breastfeeding at 1 year old had a higher risk of contracting diarrhea (57). Another study investigating the interrelationships among water access, exclusive breastfeeding, and diarrhea, involving 19 countries across Africa, including Ghana, found that children who were exclusively breastfed had 33% lower odds of diarrhea than those who were not (58). The protective effect of breastfeeding can be attributed to the presence of maternal antibodies in breast milk. Other studies have shown that oligosaccharides in breast milk act as prebiotics for the gut microbiome, promoting the growth of beneficial bacteria, which in turn reduces the incidence of diarrhea (59).

Additionally, oligosaccharides in breast milk have structures similar to specific receptors on enterocyte cell surfaces and may thus prevent the binding of certain pathogens, such as norovirus (59). Moreover, exclusive breastfeeding reduces contamination of baby food (60). As a result, several programs have been initiated in Ghana to promote exclusive breastfeeding. An example of such a program is the Start Right, Feed Right programme. This national initiative with was launched in collaboration with UN agencies focuses on encouraging breastfeeding within the 1 h after birth, encouraging exclusive breastfeeding for the first 6 months of the baby's life and introducing clean, safe solid foods at 6 months while continuing breastfeeding up to 2 years or beyond (61). Additionally, Ghana has passed the legislative Instrument 1667 (LI 1667), also known as the Breastfeeding Promotion Regulations, 2000, to regulate and limit the sale and distribution of breast milk substitutes as well as ensure they are clearly labeled with preparation directions and warning about improper preparation and other harmful consequences. Additionally, each package is expected to indicate conspicuously that breast feeding is best for the baby (62, 63). These and other initiatives that support exclusive breastfeeding have significantly reduce the incidence of childhood diarrhea. According to UNICEF Ghana, exclusive breastfeeding has reduced the risk of diarrhea 11-fold (64–66). Moreover, the National Vitamin A Supplementation Program (NVAP), which has been effective in reducing childhood mortality from cases of acute watery diarrhea. A study evaluating the effectiveness of the NVAP in northern Ghana found that mortality because of diarrhea declined from 11.1% in 1995 to 8% in 2000, and the overall severity of disease as well as hospital admissions had decreased significantly since vitamin A supplementation was implemented (67, 68).

In 2010, Ghana formally adopted the WHO/UNICEF guidelines, which recommend treating acute diarrhea in children under 5 years of age with a new, lower-osmolarity ORS and zinc (10mg for children under 6 months and 20mg for children older than 6 months, administered for 10-14 days) (69). After several programs, such as the USAID-funded SHOPS project, have worked to educate the masses on the benefits of ORS and zinc for treatment of diarrhea in children under 5 years of age, increase the availability of zinc in the local market, and train private healthcare workers on the supportive management of children with diarrhea in Ghana (69). This project increased the use of a safer and a more appropriate treatment of diarrhea and reduced the inappropriate use of antibiotics from 66.2% to 38.2% thus improving treatment outcomes (69).

These programmes, in addition to the introduction of the rotavirus vaccine into the Expanded Programme on Immunization (EPI), have helped reduce the incidence of diarrhea and its associated morbidity and mortality in Ghana. Despite this, diarrhoeal disease remains the eighth most common cause of death in children under 5 years of age as of 2021 (70). This may be because the prevalence of diarrhea is influenced not only by causative agents, but also by maternal, community, household, and individual child characteristics (71). It may therefore be necessary to tailor interventions to specific demographic groups rather than develop blanket initiatives.

## 4 Discussion

This review aims to explore the current burden of viral diarrhea in Ghana, the common viruses involved, current diagnostic practices, and public health interventions in place to address the situation. It revealed a lack of national data on the prevalence of viral diarrhea. Viral diarrhea has been estimated to be the commonest form of infectious diarrhea in children however without local data, it is difficult to make public health decisions that are relevant to the country. Additionally, the current evidence base is disproportionately focused on rotavirus, leaving important gaps in understanding the contribution of other viral pathogens.

This is concerning, as rotavirus immunization has reduced the total number of rotavirus gastroenteritis cases among children under 5 years of age. Consequently, other causes of viral diarrhea, such as norovirus are becoming relatively more prominent (72). The available studies collectively indicate a potentially high burden of viral diarrhea, although nationally representative data remain limited. This is further corroborated by studies conducted in other countries with characteristics similar to Ghana's. For instance, a meta-analysis estimated the national prevalence of rotavirus diarrhea in Nigeria to be 23% (73). In Ouagadougou, the prevalence of rotavirus has also been estimated to be 30% (74). Global studies also show this high burden. In 2016, rotavirus was estimated to be responsible for more than 258 million episodes of diarrhea among children under 5 years of age and 128,500 deaths in this age group, with 104,733 of these deaths occurring in Sub-Saharan Africa (6). It is therefore essential that further studies be conducted to ascertain the actual burden of viral diarrhea in the country. This will be helpful in healthcare planning and the equitable allocation of resources. Prevalence studies would provide information on regional and district hotspots, helping deliver targeted interventions rather than blanket solutions.

This review also explored the various viruses usually implicated in diarrhea in children under 5 years of age in Ghana. It showed a predominance of rotavirus gastroenteritis over other viruses. This finding is unsurprising, as Rotavirus is the leading cause of dehydrating diarrhea in children under 5 years of age globally (72). This is further supported by a meta-analysis investigating the aetiological causes of diarrhea in sub-Saharan Africa, which found that rotavirus was the most common cause at 29.2% (75). Nevertheless, recent studies suggest an increasing contribution of norovirus gastroenteritis, while the prevalence of rotavirus gastroenteritis appears to be declining (72). In some more developed countries, noroviruses has emerged as a leading cause of diarrhea in children under 5 years of age (76). Although these epidemiological shifts have been observed following the introduction of rotavirus vaccination programmes, current evidence is insufficient to establish a direct causal relationship between rotavirus vaccination and changes in norovirus epidemiology (72, 76). Given the growing public health importance of norovirus and the absence of a licensed vaccine, continued surveillance and further research into norovirus vaccine development remain warranted.

Additionally, genotype combinations of rotavirus that were previously uncommon have gained a resurgence in their prevalence. This is concerning, as the rotavirus vaccine was designed to target specific virus genotypes (77, 78). A surge in the prevalence of uncommon rotavirus genotypes could undo the impact of rotavirus vaccination and increase rotavirus gastroenteritis morbidity and mortality worldwide. It is thus important to introduce a robust surveillance system to monitor the circulating genotypes in the community. In Ghana, a disparity has been observed in the impact of the rotavirus vaccination between the northern and southern regions. Apart from numerous socio-economic factors, such as poverty and educational background, some studies have postulated that maternal and child characteristics, including maternal immunity, infant gut microbiome, and the timing of first infections, play a role (79–81). However, these principles are not fully understood. Further research is therefore essential to fully understand the predictors of vaccine response in lower-income countries and improve the impact of the rotavirus vaccine in these regions.

This study also revealed that Ghana, like many other countries, relies predominantly on WHO/UNICEF guidelines for the management of acute diarrhea in children. While these guidelines offer valuable clues to the likely causative agents and help direct appropriate treatment, they do not establish the definitive etiology, leaving clinicians to base management decisions on informed assumptions rather than confirmed diagnoses (82). Although Ghana has established diagnostic capacity for viral diarrhea—primarily through rapid antigen assays and molecular platforms such as PCR—these resources are disproportionately concentrated in the capital, research laboratories and a handful of regional hospitals. As a result, most rural and district hospitals lack in-house capability for pathogen-specific diagnosis. Even in facilities where laboratory services exist, they are often oriented toward bacterial culture rather than viral detection (83).

Consequently, many patients must be referred to distant regional or municipal centers for confirmatory testing, a process that is frequently interrupted by logistical barriers. Weak sample transport systems, unreliable cold chain maintenance, poor road infrastructure leading to prolonged transit times, and erratic electricity supply all contribute to sample degradation before arrival at referral laboratories. In addition, the limited number of molecular diagnostic machines serving multiple facilities, combined with slow transport of stool samples, results in long turnaround times. This delays case confirmation, impedes early detection, and weakens surveillance for emerging viral pathogens (84).

To combat this, many healthcare workers rely mainly on clinical evaluation to diagnose patients. Treatment guidelines based on clinical symptoms are generally effective and important in resource-limited countries like Ghana. However, many healthcare professionals struggle to distinguish between viral and bacterial diarrhea, as symptoms often overlap (82). This often leads to inappropriate antibiotic prescribing, increasing antimicrobial resistance (AMR) in the country (85). In Ghana, AMR is a significant threat associated with an increased mortality for patients. It is estimated that since 1990, about 5000 deaths anaually are attributed to AMR in Ghana (86). In 2021, AMR was found to be directly responsible for about 5,000 deaths and indirectly associated with 23,100 fatalities most of which occurred in children under 5 years (86, 87). In addition to increased mortality rates, AMR is also associated with increased healthcare cost. Due to antibiotic missue, many patients contract infections that are already resistance to the common and relatively cheaper antibiotics in the society. Prescribers therefore have to recommend costly antibiotics. Also, some patients are unable to afford the costs of these medications and even in situations where they can, these antibiotics are associated with more adverse effects and complicatiions which may lead to longer hospital stays and poorer health outcomes. It is important to ensure that antibiotics are indicated before it is prescribed (88, 89).

Ghana's limited capacity to diagnose viral diarrhea also impairs disease surveillance in the country. Ghana uses the Integrated Disease Surveillance and Report system (IDSR) which depends significantly on information it receives from Community-based Health Planning and Service (CHPS) and health centers (83). However, lower-level clinics and community health centers often lack the diagnostic capacity needed to distinguish between bacterial, parasitic, and viral causes of acute gastroenteritis. This causes health care workers to diagnose and treat diarrheal cases as unspecified acute watery diarrhea (90).

As such, the true burden of viral pathogens remains unrecognized making decisions on resource allocation and other public health initiatives difficult. Additionally, empiric management and delays in laboratory diagnosis impede real time surveillance and thus an uptick in viral diarrhea in the community may snowball into an outbreak before it is detected and managed. Furthermore, this limited detection capacity makes it difficult to track new emerging rotavirus genotypes making evaluation of rotavirus vaccine efficacy difficult (84). It is thus essential to move toward more pathogen-specific diagnostic testing in the management of diarrhea.

## 5 Strengths and limitations

This scoping review offers a comprehensive overview of viral diarrhea research in Ghana, consolidating evidence across multiple databases and diverse study designs while adhering to PRISMA-ScR reporting standards.

Nevertheless, several limitations are inherent to the methodology and available literature. Scoping reviews do not typically include formal quality appraisal, which restricts the ability to assess the robustness of individual studies. The included evidence is also subject to publication bias and variability in methodological rigor, with many studies characterized by small sample sizes, and limited generalizability. Finally, reliance on published and accessible data means that gaps in surveillance and reporting may have constrained the completeness of the evidence base.

## 6 Conclusion

Ghana has made significant strides in the prevention and management of diarrhoeal diseases, particularly those of viral origin. Despite progress in reducing viral diarrhoeal disease burden in Ghana, significant challenges remain, including limited diagnostic capacity, uneven disease burden across regions, and insufficient surveillance of non-rotavirus pathogens. Strengthening surveillance systems, improving access to diagnostic testing, and implementing context-specific prevention strategies will be essential for reducing the burden of viral diarrhea in Ghana. The emergence of new rotavirus genotypes and the observed increases in norovirus detection following introduction of the rotavirus vaccine suggest a dynamic nature of pathogen ecology and the need for ongoing nationwide surveillance.

While viral diarrhea is generally managed according to standardized guidelines, healthcare facilities need to implement diagnostic tests that accurately identify specific etiologies. This would not only reduce inappropriate antibiotic use in cases of viral diarrhea but also inform vaccine development and guide evidence-based prevention strategies. Ultimately, strengthening diagnostic capacity and scaling up surveillance efforts will be pivotal in addressing the evolving challenges of viral diarrhea in Ghana.

## Data Availability

The original contributions presented in the study are included in the article/Supplementary material, further inquiries can be directed to the corresponding authors.

## References

[1] World Health Organization. World Health Organization. (2024). Diarrhoea. Available online at: https://www.who.int/health-topics/diarrhoea#tab=tab_1 (Accessed February 7, 2025).

[2] Platts-Mills JA Liu J Rogawski ET Kabir F Lertsethtakarn P Siguas M . Use of quantitative molecular diagnostic methods to assess the aetiology, burden, and clinical characteristics of diarrhoea in children in low-resource settings: a reanalysis of the MAL-ED cohort study. Lancet Glob Health. (2018) 6:e1309. doi: 10.1016/S2214-109X(18)30349-8 30287127 PMC6227251

[3] Akuffo R Armah G Clemens M Kronmann KC Jones AH Agbenohevi P . Prevalence of enteric infections among hospitalized patients in two referral hospitals in Ghana. BMC Res Notes. (2017) 10:292. doi: 10.1186/s13104-017-2621-x 28716138 PMC5514524

[4] Arksey H O'Malley L. Scoping studies: towards a methodological framework. Int J Soc Res Methodol. (2005) 8:19–32. doi: 10.1080/1364557032000119616

[5] Statistical Service G. Ghana Demographic and Health Survey. (2022). Available online at: http://www.DHSprogram.com (Accessed February 12, 2025).

[6] Troeger C Khalil IA Rao PC Cao S Blacker BF Ahmed T . Rotavirus Vaccination and the global burden of rotavirus diarrhea among children younger than 5 years. JAMA Pediatr. (2018) 172:958–65. doi: 10.1001/jamapediatrics.2018.1960 30105384 PMC6233802

[7] Dzotsi EK Dongdem AZ Boateng G Antwi L Owusu-Okyere G Nartey DB . Surveillance of bacterial pathogens of diarrhoea in two selected sub metros within the accra metropolis. Ghana Med J. (2015) 49:65. doi: 10.4314/gmj.v49i2.1 26339088 PMC4549830

[8] Bandoh DA Dwomoh D Yirenya-Tawiah D Kenu E Dzodzomenyo M. Prevalence and correlates of diarrhoea among children under five in selected coastal communities in Ghana. J Health Popul Nutr. (2024) 43:95. doi: 10.1186/s41043-024-00582-8 38926857 PMC11210189

[9] Asare EO Warren JL Pitzer VE. Spatiotemporal patterns of diarrhea incidence in ghana and the impact of meteorological and socio-demographic factors. Front Epidemiol. (2022) 2:871232. doi: 10.3389/fepid.2022.871232 35822109 PMC9272077

[10] Armah G Pringle K Enweronu-Laryea CC Ansong D Mwenda JM Diamenu SK . Impact and effectiveness of monovalent rotavirus vaccine against severe rotavirus diarrhea in Ghana. Clin Infect Dis. (2016) 62:S200–7. doi: 10.1093/cid/ciw014 27059357 PMC7962386

[11] Enweronu-Laryea CC Sagoe KWC Glover-Addy H Asmah RH Mingle JA Armah GE. Prevalence of severe acute rotavirus gastroenteritis and intussusceptions in Ghanaian children under 5 years of age. J Infect Dev Countries. (2011) 6:148–55. doi: 10.3855/jidc.1667 22337844

[12] Binka EB Vermund SH Armah GE. Rotavirus diarrhea among children less than 5 years of age in urban Ghana. Pediatr Infect Dis J. (2011) 30:716–8. doi: 10.1097/INF.0b013e318223bd85 21646938 PMC3149899

[13] Damanka S Adiku TK Armah GE Rodrigues O Donkor ES Nortey D . Rotavirus infection in children with diarrhea at Korle-Bu teaching hospital, Ghana. Jpn J Infect Dis. (2016) 69:331–4. doi: 10.7883/yoken.JJID.2014.407 26370427

[14] Adu GA Amegah KE Addo HO Andoh T Duvor F Antwi G . Reduction in diarrhea cases following implementation of COVID-19 hand hygiene interventions in Ghana: a causal impact analysis. PLoS ONE. (2024) 19:e0309202. doi: 10.1371/journal.pone.0309202 39208316 PMC11361678

[15] Ghana Statistical Service (GSS) and ICF. Ghana Demographic and Health Survey 2022: Key Indicators Report. Accra, Ghana, and Rockville, Maryland, USA: GSS and ICF. Accra, Ghana, and Rockville, Maryland, USA (2023) Available online at: https://dhsprogram.com/pubs/pdf/PR149/PR149.pdf (Accessed March 19, 2025).

[16] Troeger C Forouzanfar M Rao PC Khalil I Brown A Reiner RC . Estimates of global, regional, and national morbidity, mortality, and aetiologies of diarrhoeal diseases: a systematic analysis for the Global Burden of Disease Study 2015. Lancet Infect Dis. (20171) 17:909. doi: 10.1016/S1473-3099(17)30276-1 PMC5589208 28579426

[17] Tate JE Burton AH Boschi-Pinto C Parashar UD Agocs M Serhan F . Global, regional, and national estimates of rotavirus mortality in children < 5 years of age, 2000–2013. Clin Infect Dis. (2016) 62:S96–105. doi: 10.1093/cid/civ1013 27059362 PMC11979873

[18] Kyu HH Vongpradith A Dominguez RMV Ma J Albertson SB Novotney A . Global, regional, and national age-sex-specific burden of diarrhoeal diseases, their risk factors, and aetiologies, 1990–2021, for 204 countries and territories: a systematic analysis for the Global Burden of Disease Study 2021. Lancet Infect Dis. (2025). 10.1016/S1473-3099(24)00691-1 PMC12018300 39708822

[19] Kombat MY Kushitor SB Sutherland EK Boateng MO Manortey S. Prevalence and predictors of diarrhea among children under five in Ghana. BMC Public Health. (2024) 24:1–11. doi: 10.1186/s12889-023-17575-7 38212722 PMC10782682

[20] UNICEF. Diarrhoea - UNICEF DATA. (2024). Available online at: https://data.unicef.org/topic/child-health/diarrhoeal-disease/ (Accessed May 16, 2025).

[21] Appiah-Effah E Sagoe G Afful KM Yamoah-Antwi D. Assessment of the health impacts of WASH interventions in disaster-prone communities in three regions of Northern Ghana. Afr J Environ Sci Technol. (2020) 14:269–80. doi: 10.5897/AJEST2019.2802

[22] Reither K Ignatius R Weitzel T Seidu-Korkor A Anyidoho L Saad E . Acute childhood diarrhoea in northern Ghana: epidemiological, clinical and microbiological characteristics. BMC Infect Dis. (2007) 7:104. doi: 10.1186/1471-2334-7-104 17822541 PMC2018704

[23] Enweronu-Laryea CC Sagoe KW Damanka S Lartey B Armah GE. Rotavirus genotypes associated with childhood severe acute diarrhoea in southern Ghana: a cross-sectional study. Virol J. (2013) 10:1–6. doi: 10.1186/1743-422X-10-287 24034588 PMC3848793

[24] Enweronu-Laryea CC Boamah I Sifah E Diamenu SK Armah G. Decline in severe diarrhea hospitalizations after the introduction of rotavirus vaccination in Ghana: a prevalence study. BMC Infect Dis. (2014) 14:1–6. doi: 10.1186/1471-2334-14-431 25100574 PMC4132910

[25] Bawa FK Mutocheluh M Dassah SD Ansah P Oduro AR. Genetic diversity of rotavirus infection among young children with diarrhoea in the Kassena-Nankana Districts of Northern Ghana: a seasonal cross-sectional survey. PAMJ. (2023) 44:148. doi: 10.11604/pamj.2023.44.148.36783 37396694 PMC10311230

[26] Armah GE Mingle JAA Dodoo AK Anyanful A Antwi R Commey J . Seasonality of rotavirus infection in Ghana. Ann Trop Paediatr. (1994) 14:223–9. doi: 10.1080/02724936.1994.11747721 7825996

[27] Silva PA Stark K Mockenhaupt FP Reither K Weitzel T Ignatius R . Molecular characterization of enteric viral agents from children in northern region of Ghana. J Med Virol. (2008) 80:1790–8. doi: 10.1002/jmv.21231 18712819

[28] Armah GE Hoshino Y Santos N Binka F Damanka S Adjei R . The Global spread of rotavirus G10 strains: detection in Ghanaian children hospitalized with diarrhea. J Infect Dis. (2010) 202:S231–8. doi: 10.1086/653572 20684709 PMC2954461

[29] Enweronu-Laryea CC Sagoe KWC Mwenda JM Armah GE. Severe acute rotavirus gastroenteritis in children less than 5 years in southern Ghana: 2006-2011. Pediatr Infect Dis J. (2014) 33 Suppl.:S9–S13. doi: 10.1097/INF.0000000000000045 24343622

[30] Ugboko HU Nwinyi OC Oranusi SU Oyewale JO. Childhood diarrhoeal diseases in developing countries. Heliyon. (2020) 6:e03690. doi: 10.1016/j.heliyon.2020.e03690 32322707 PMC7160433

[31] Nemeth V Pfleghaar N. Diarrhea. Pocket Guide to IBD, 2nd Edition. Boca Raton, FL:CRC Press (2022) p. 41–50.

[32] Mokomane M Kasvosve I Melo E de Pernica JM. Goldfarb DM. The global problem of childhood diarrhoeal diseases: emerging strategies in prevention and management. Ther Adv Infect Dis. (2017) 5:29–43. doi: 10.1177/2049936117744429 29344358 PMC5761924

[33] Existing WHO guidelines for preventing and treating diarrhoea in children. (2010). Available online at: https://www.ncbi.nlm.nih.gov/books/NBK305340/ (Accessed February 18, 2025).

[34] Standard Treatment Guidelines. (2017). Available online at: www.ghndp.org (Accessed February 18, 2025).

[35] Glass RI Tate JE Jiang B Parashar U. The rotavirus vaccine story: from discovery to the eventual control of rotavirus disease. J Infect Dis. (2021) 224:S331. doi: 10.1093/infdis/jiaa598 34590142 PMC8482027

[36] Adjei MR Ofori Amoah J Bonsu G Okine R Mohammed NT Amponsa-Achiano K . Has Ghana's rotavirus vaccine switch met programmatic expectations? an analysis of national surveillance data; 2018–2022. Open Forum Infect Dis. (2024) 11:ofae539. doi: 10.1093/ofid/ofae539 39364172 PMC11447473

[37] Statistical Service G. Demographic and Health Survey. Reston, ICF Macro (2008).

[38] Statistical Service Accra G. Ghana Demographic and Health Survey. (2014). 2015. Available online at: http://www.DHSprogram.com (Accessed February 10, 2025).

[39] Lartey BL Damanka S Dennis FE Enweronu-Laryea CC Addo-Yobo E Ansong D . Rotavirus strain distribution in Ghana pre- and post- rotavirus vaccine introduction. Vaccine. (2018) 36:7238–42. doi: 10.1016/j.vaccine.2018.01.010 29371014 PMC11345725

[40] Enweronu-Laryea CC Armah G Sagoe KW Ansong D Addo-Yobo E Diamenu SK . Sustained impact of rotavirus vaccine introduction on rotavirus gastroenteritis hospitalizations in children < 5 years of age, Ghana, 2009–2016. Vaccine. (2018) 36:7131–4. doi: 10.1016/j.vaccine.2018.02.058 29752020 PMC8848125

[41] Asare EO Al-Mamun MA Armah GE Lopman BA Parashar UD Binka F . Modeling of rotavirus transmission dynamics and impact of vaccination in Ghana. Vaccine. (2020) 38:4820–8. doi: 10.1016/j.vaccine.2020.05.057 32513513 PMC8290434

[42] Ofosu-Appiah LH Negoro M Amexo JX Amelor DK Tonto PB Laryea DO . Clinical impact and genetic analysis of enteric viruses associated with acute gastroenteritis in greater Accra, Ghana: a comprehensive study of five viruses. J Med Virol. (2025) 97:e70216. doi: 10.1002/jmv.70216 39935201

[43] Letsa V Damanka S Dennis F Lartey B Armah GE Betrapally N . Distribution of rotavirus genotypes in the postvaccine introduction era in Ashaiman, Greater Accra Region, Ghana, 2014-2016. J Med Virol. (2019) 91:2025–8. doi: 10.1002/jmv.25542 31286526 PMC12001282

[44] Doan YH Dennis FE Takemae N Haga K Shimizu H Appiah MG . Emergence of Intergenogroup Reassortant G9P[4] Strains Following Rotavirus Vaccine Introduction in Ghana. Viruses. (2023) 15:2453. doi: 10.3390/v15122453 38140694 PMC10747750

[45] Steele AD Armah GE Mwenda JM Kirkwood CD. The full impact of rotavirus vaccines in africa has yet to be realized. Clin Infect Dis. (2023) 76:S1–4. doi: 10.1093/cid/ciad017 37074434 PMC10116555

[46] Otieno GP Bottomley C Khagayi S Adetifa I Ngama M Omore R . Impact of the introduction of rotavirus vaccine on hospital admissions for diarrhea among children in Kenya: a controlled interrupted time-series analysis. Clin Infect Dis. (2019) 70:2306. doi: 10.1093/cid/ciz912 31544211 PMC7245159

[47] Varghese T Kang G Steele AD. Understanding rotavirus vaccine efficacy and effectiveness in countries with high child mortality. Vaccines. (2022) 10:346. doi: 10.3390/vaccines10030346 35334978 PMC8948967

[48] Mujuru HA Yen C Nathoo KJ Gonah NA Ticklay I Mukaratirwa A . Reduction in diarrhea- and rotavirus-related healthcare visits among children < 5 years of age following national rotavirus vaccine introduction in Zimbabwe. Pediatr Infect Dis J. (2017) 36:995. doi: 10.1097/INF.0000000000001648 28640001 PMC5600692

[49] Sanneh B Papa Sey A Shah M Tate J Sonko M Jagne S . Impact of pentavalent rotavirus vaccine against severe rotavirus diarrhoea in The Gambia. Vaccine. (2018) 36:7179–84. doi: 10.1016/j.vaccine.2018.02.091 29544688 PMC6816024

[50] Bonkoungou IJO Aliabadi N Leshem E Kam M Nezien D Drabo MK . Impact and effectiveness of pentavalent rotavirus vaccine in children < 5 years of age in Burkina Faso. Vaccine. (2017) 36:7170. doi: 10.1016/j.vaccine.2017.12.056 29290478 PMC9237810

[51] Diop A Thiongane A Mwenda JM Aliabadi N Sonko MA Diallo A . Impact of rotavirus vaccine on acute gastroenteritis in children under 5 years in Senegal: Experience of sentinel site of the Albert Royer Children's Hospital in Dakar. Vaccine. (2018) 36:7192–7. doi: 10.1016/j.vaccine.2017.10.061 29162319 PMC5959761

[52] WHO. Preventing diarrhoea through better water, sanitation and hygiene: exposures and impacts in low- and middle-income countries. Geneva: World Health Organization (2014). p. 1–48. Available online at: http://www.who.int/water_sanitation_health/publications/preventing-diarrhoea/en/

[53] World Health Organisation. Diarrhoeal disease. (2024). Available online at: https://www.who.int/news-room/fact-sheets/detail/diarrhoeal-disease (Accessed February 17, 2025).

[54] Bauza V Ye W Liao J Majorin F Clasen T. Interventions to improve sanitation for preventing diarrhoea. Cochrane Database Syst Rev. (2023) 2023:CD013328. doi: 10.1002/14651858.CD013328.pub2 36697370 PMC9969045

[55] Gros C. UNICEF. A New Game to Teach Children Essential Hygiene Practices | UNICEF Office of Innovation. (2016). Available online at: https://www.unicef.org/innovation/stories/new-game-teach-children-essential-hygiene-practices (Accessed March 12, 2025).

[56] Handwashing with Ananse - Research Projects - Engagement Lab. Available online at: https://engagementlab.work/research/projects/handwashing-with-ananse/ (Accessed June 13, 2026).

[57] Ogbo FA Agho K Ogeleka P Woolfenden S Page A Eastwood J . Infant feeding practices and diarrhoea in sub-Saharan African countries with high diarrhoea mortality. PLoS ONE. (2017) 12. doi: 10.1371/journal.pone.0171792 28192518 PMC5305225

[58] Apanga PA Weber AM Darrow LA Riddle MS Tung WC Liu Y . The interrelationship between water access, exclusive breastfeeding and diarrhea in children: a cross-sectional assessment across 19 African countries. J Glob Health. (2021) 11:1–9. doi: 10.7189/jogh-11-04001 33828842 PMC8005312

[59] Turin CG Ochoa TJ. The role of maternal breast milk in preventing infantile diarrhea in the developing world. Curr Trop Med Rep. (2014) 1:97–105. doi: 10.1007/s40475-014-0015-x 24883263 PMC4036098

[60] Webb C Cabada MM. A review on prevention interventions to decrease diarrheal diseases' burden in children. Curr Trop Med Rep. (2018) 5:31–40. doi: 10.1007/s40475-018-0134-x

[61] UNICEF-Ghana. First Lady of Republic of Ghana launches one-year infant and young child feeding campaign. (2020). Available online at: https://www.unicef.org/ghana/press-releases/first-lady-republic-ghana-launches-one-year-infant-and-young-child-feeding-campaign (Accessed June 13, 2026).

[62] World Health Organization. Ghana Health Service and its Health Partners engage the media during Breastfeeding Week | WHO | Regional Office for Africa. (2018). Available online at: https://www.afro.who.int/news/ghana-health-service-and-its-health-partners-engage-media-during-breastfeeding-week (Accessed June 13, 2026).

[63] Alabi G Alabi J Moses IS. Effects of the law on the marketing of infant foods in Ghana. Int Bus Econ Res J. (2007) 6. doi: 10.19030/iber.v6i6.3378

[64] World Health Organization. Let's support mothers to breastfeed as long as they want- First Lady of Ghana advocates | WHO | Regional Office for Africa. (2020). Available online at: https://www.afro.who.int/news/lets-support-mothers-breastfeed-long-they-want-first-lady-ghana-advocates (Accessed June 13, 2026).

[65] UNICEF. Why Exclusive Breastfeeding Matters | UNICEF Ghana. (2025). Available online at: https://www.unicef.org/ghana/stories/why-exclusive-breastfeeding-matters (Accessed June 13, 2026).

[66] Nii Okai Aryeetey R Antwi CL. Re-assessment of selected baby-friendly maternity facilities in Accra, Ghana. Int Breastfeed J. (2013) 8:1. doi: 10.1186/1746-4358-8-15 24216173 PMC3832220

[67] Ross DA Dollimore N Smith PG Kirkwood BR Arthur P Morris SS . Vitamin A supplementation in northern Ghana: effects on clinic attendances, hospital admissions, and child mortality. Lancet. (1993) 342:7–12. doi: 10.1016/0140-6736(93)91879-Q 8100345

[68] David P. Evaluating the vitamin A Supplementation programme in northern ghana: has it contributed to improved child survival? Boston. (2003).

[69] El-Khoury M Banke K Sloane P. Improved childhood diarrhea treatment practices in Ghana: A pre-post evaluation of a comprehensive private-sector program. Glob Health Sci Pract. (2016) 4:264–75. doi: 10.9745/GHSP-D-16-00021 27353619 PMC4982250

[70] Dattani S Spooner F Ritchie H Roser M. Diarrheal diseases. Our World in Data. (2023). Retrieved from: https://ourworldindata.org/diarrheal-diseases (Accessed June 13, 2026).

[71] Kumi-Kyereme A Amo-Adjei J. Household wealth, residential status and the incidence of diarrhoea among children under-five years in Ghana. J Epidemiol Glob Health. (2015) 6:131. doi: 10.1016/j.jegh.2015.05.001 26070430 PMC7320473

[72] LeClair CE McConnell KA. Rotavirus. StatPearls. Treasure Island, FL: StatPearls Publishing (2023). 32644377

[73] Digwo D Chidebelu P Ugwu K Adediji A Farkas K Chigor V. Prevalence and relative risk of Rotavirus Gastroenteritis in children under five years in Nigeria: a systematic review and meta-analysis. Pathog Glob Health. (2023) 117:24–35. doi: 10.1080/20477724.2022.2043223 35249468 PMC9848363

[74] Bonkoungou IJO Haukka K Österblad M Hakanen AJ Traoré AS Barro N . Bacterial and viral etiology of childhood diarrhea in Ouagadougou, Burkina Faso. BMC Pediatr. (2013) 13:36. doi: 10.1186/1471-2431-13-36 23506294 PMC3616825

[75] Oppong TB Yang H Amponsem-Boateng C Kyere EKD Abdulai T Duan G . Enteric pathogens associated with gastroenteritis among children under 5 years in sub-Saharan Africa: a systematic review and meta-analysis. Epidemiol Infect. (2020) 148:e64. doi: 10.1017/S0950268820000618 32115003 PMC7118358

[76] Capece G Tobin EH Gignac E. Norovirus. Encyclopedia of Food Safety, 2nd Edition. Elsevier: Academic Press (2025) 1–4:V2-439-V2-449. doi: 10.1016/B978-0-12-822521-9.00058-7

[77] Ruiz-Palacios GM Pérez-Schael I Velázquez FR Abate H Breuer T Clemens SC . Safety and Efficacy of an Attenuated Vaccine against Severe Rotavirus Gastroenteritis. N Engl J Med. (2006) 354:11–22. doi: 10.1056/NEJMOA052434 16394298

[78] Ward RL Clark HF Offit PA. Influence of potential protective mechanisms on the development of live rotavirus vaccines. J Infect Dis. (2010) 202:S72–S79. doi: 10.1086/653549 20684721

[79] Armah GE Kapikian AZ Vesikari T Cunliffe N Jacobson RM Burlington DB . Efficacy, immunogenicity, and safety of two doses of a tetravalent rotavirus vaccine RRV-TV in Ghana with the first dose administered during the neonatal period. J Infect Dis. (2013) 208:423–31. doi: 10.1093/infdis/jit174 23599316 PMC3699001

[80] Chilengi R Simuyandi M Beach L Mwila K Becker-Dreps S Emperador DM . Association of maternal immunity with rotavirus vaccine immunogenicity in zambian infants. PLoS ONE. (2016) 11:e0150100. doi: 10.1371/journal.pone.0150100 26974432 PMC4790930

[81] Harris VC Armah G Fuentes S Korpela KE Parashar U Victor JC . Significant Correlation between the Infant Gut Microbiome and Rotavirus Vaccine Response in Rural Ghana. J Infect Dis. (2017) 215:34–41. doi: 10.1093/infdis/jiw518 27803175 PMC5225256

[82] Akhondi H Goldin J Simonsen KA. Bacterial Diarrhea - PubMed. (2025). Available online at: https://pubmed.ncbi.nlm.nih.gov/31869107/ 7 (Accessed June 13, 2026).

[83] Adachi M Taniguchi K Hori H Mizutani T Ishizaka A Ishikawa K . Strengthening surveillance in Ghana against public health emergencies of international concern. Trop Med Health. (2022) 50:1. doi: 10.1186/s41182-022-00473-w 36307880 PMC9617445

[84] World Health Organization. Sustaining vaccine gains through stronger rotavirus surveillance in Ghana | WHO | Regional Office for Africa. (2026). Available online at: https://www.afro.who.int/countries/ghana/news/sustaining-vaccine-gains-through-stronger-rotavirus-surveillance-ghana (Accessed June 13, 2026).

[85] Yevutsey SK Buabeng KO Aikins M Anto BP Biritwum RB Frimodt-Møller N . Situational analysis of antibiotic use and resistance in Ghana: Policy and regulation. BMC Public Health. (2017) 17:1–7. doi: 10.1186/s12889-017-4910-7 29169340 PMC5701378

[86] Institute for Health Metrics and Evaluation (IHME). The burden of antimicrobial resistance (AMR) in Ghana. Seattle, WA: IHME (2024). Available online at: https://www.healthdata.org/sites/default/files/2023-09/Ghana.pdf (Accessed June 13, 2026).

[87] World Health Organization. Ghana Leads Africa in Advancing a People-Centred Approach to Address Antimicrobial Resistance | WHO | Regional Office for Africa. (2026). Available online at: https://www.afro.who.int/countries/ghana/news/ghana-leads-africa-advancing-people-centred-approach-address-antimicrobial-resistance (Accessed June 13, 2026).

[88] Otieku E Fenny AP Labi AK Ofori AO Kurtzhals JAL Enemark U. Attributable patient cost of antimicrobial resistance: a prospective parallel cohort study in two public teaching hospitals in Ghana. Pharmacoecon Open. (2023) 7:257. doi: 10.1007/s41669-022-00385-9 36692621 PMC10043073

[89] Fenny AP Otieku E Amoah RO Jejeti M Labi AK Hedidor GK . Attributable societal cost of antimicrobial resistance in Ghana: a microsimulation study focusing on sociodemographic groups. BMJ Open. (2026) 16:e111045. doi: 10.1136/bmjopen-2025-111045 41638750 PMC12878375

[90] Adokiya MN Awoonor-Williams JK Beiersmann C Müller O. The integrated disease surveillance and response system in northern Ghana: challenges to the core and support functions. BMC Health Serv Res. (2015) 15:1. doi: 10.1186/s12913-015-0960-7 26216356 PMC4515924

[91] Armah GE Gallimore CI Binka FN Asmah RH Green J Ugoji U . Characterisation of norovirus strains in rural Ghanaian children with acute diarrhoea. J Med Virol. (2006) 78:1480–5. doi: 10.1002/jmv.20722 16998875

[92] Lartey BL Quaye O Damanka SA Agbemabiese CA Armachie J Dennis FE . Understanding pediatric norovirus epidemiology: a decade of study among ghanaian children. Viruses. (2020) 12:1321. doi: 10.3390/v12111321 33217894 PMC7698731

[93] Krumkamp R Sarpong N Schwarz NG Adelkofer J Loag W Eibach D . Gastrointestinal infections and diarrheal disease in Ghanaian infants and children: an outpatient case-control study. PLoS Negl Trop Dis. (2015) 9:e0003568. doi: 10.1371/journal.pntd.0003568 25738935 PMC4349824

[94] Ashie GK Mutocheluh M Owusu M Kwofie TB Akonor S Narkwa PW . Microbial pathogens associated with acute childhood diarrhoea in Kumasi, Ghana. BMC Res Notes. (2017) 10:1–7. doi: 10.1186/s13104-017-2578-9 28693616 PMC5504554

[95] Thystrup C Majowicz SE Kitila DB Desta BN Fayemi OE Ayolabi CI . Etiology-specific incidence and mortality of diarrheal diseases in the African region: a systematic review and meta-analysis. BMC Public Health. (20241) 24:1–26. doi: 10.1186/s12889-024-19334-8 38997671 PMC11241906

[96] Osei FB Stein A. Spatial variation and hot-spots of district level diarrhea incidences in Ghana: 2010-2014. BMC Public Health. (2017) 17:1–10. doi: 10.1186/s12889-017-4541-z 28673274 PMC5496362

[97] Hori H Akpedonu P Armah G Aryeetey M Yartey J Kamiya H . Enteric pathogens in severe forms of acute gastroenteritis in Ghanaian children. Pediatrics International. (1996) 38:672–6. doi: 10.1111/j.1442-200X.1996.tb03729.x 9002307

[98] Operario DJ Platts-Mills JA Nadan S Page N Seheri M Mphahlele J . Etiology of severe acute watery diarrhea in children in the global rotavirus surveillance network using quantitative polymerase chain reaction. J Infect Dis. (2017) 216:220–7. doi: 10.1093/INFDIS/JIX294 28838152 PMC5853801

[99] Mizutani T Aboagye SY Ishizaka A Afum T Mensah GI Asante-Poku A . Gut microbiota signature of pathogen-dependent dysbiosis in viral gastroenteritis. Sci Rep. (2021) 11:1. doi: 10.1038/s41598-021-93345-y 34230563 PMC8260788

[100] Cohen AL Platts-Mills JA Nakamura T Operario DJ Antoni S Mwenda JM . Aetiology and incidence of diarrhoea requiring hospitalisation in children under 5 years of age in 28 low-income and middle-income countries: findings from the Global Pediatric Diarrhea Surveillance network. BMJ Glob Health. (2022) 7:e009548. doi: 10.1136/bmjgh-2022-009548 36660904 PMC9445824

[101] Heinemann M Strauchs C Lütgehetmann M Aepfelbacher M Klupp EM Owusu-Dabo E . Polymicrobial enteric infections in African infants with diarrhoea—results from a longitudinal prospective case–control study. Clinic Microbiol Infect. (20211) 27:1792–8. doi: 10.1016/j.cmi.2021.03.020 33813114

[102] Armah GE Steele AD Binka FN Esona MD Asmah RH Anto F . Changing patterns of rotavirus genotypes in Ghana: Emergence of human rotavirus G9 as a major cause of diarrhea in children. J Clin Microbiol. (2003) 41:2317–22. doi: 10.1128/JCM.41.6.2317-2322.2003 12791843 PMC156506

[103] Navrongo T Research Group R. Incidence and risk factors of paediatric rotavirus diarrhoea in northern Ghana. Trop Med Int Health. (2003) 8:840–6. doi: 10.1046/j.1365-3156.2003.01097.x 12950670

[104] Armah GE Steele AD Esona MD Akran VA Nimzing L Pennap G. Diversity of rotavirus strains circulating in west Africa from 1996 to 2000. J Infect Dis. (2010) 202 Suppl:2317–22. doi: 10.1086/653571 20684720

[105] Asmah RH Green J Armah GE Gallimore CI Gray JJ Iturriza-Gómara M . Rotavirus G and P genotypes in rural Ghana. J Clin Microbiol. (2001) 39:1981–4. doi: 10.1128/JCM.39.5.1981-1984.2001 11326029 PMC88064

[106] George E Armah Cara T Pager Richard H. Asmah, Francis R. Anto, Abraham Rexdford Oduro, Fred Binka, et al. Prevalence of unusual human rotavirus strains in Ghanaian children. (2001). p. 67–71. Available online at: https://onlinelibrary.wiley.com/doi/epdf/10.1002/1096-9071%28200101%2963%3A1%3C67%3A%3AAID-JMV1010%3E3.0.CO%3B2-T (Accessed April 7, 2026).

