# Supplementary material for: Viral diarrhea in Ghana: a scoping review of disease burden, epidemiology, and control strategies
Source: Front Public Health. 2026 Jul 29;14:1840821. doi: 10.3389/fpubh.2026.1840821 (PMC13463948; doi:10.3389/fpubh.2026.1840821)
Supplement: Supplementary file 1 [file Table_1.docx]

**Supplementary Materials.**

**Search Strings**

| **Database** | **Keywords** |
| --- | --- |
| **PubMed (206)** | ("Diarrhea"[Mesh] OR diarrh* [tiab] OR "gastroenteritis"[tiab])  AND ("Viruses"[Mesh] OR virus[tiab] OR viral[tiab] OR rotavirus[tiab] OR norovirus[tiab] OR adenovirus[tiab] OR astrovirus[tiab] OR sapovirus[tiab])  AND ("Ghana"[Mesh] OR Ghana[tiab] OR Ghana)  ("Diarrhea"[Mesh] OR diarrh* [tiab])  AND ("Viruses"[Mesh] OR rotavirus[tiab] OR norovirus[tiab] OR adenovirus[tiab] OR astrovirus[tiab])  AND ("Epidemiology"[Mesh] OR epidemiolog*[tiab] OR burden[tiab] OR prevalence[tiab] OR incidence[tiab] OR surveillance[tiab] OR "public health"[tiab] OR control[tiab] OR intervention[tiab] OR sanitation[tiab] OR "water supply"[Mesh])  AND ("Ghana"[Mesh] OR Ghana[tiab] OR Ghana) |
| **Scopus (247)** | TITLE-ABS-KEY ( diarrhea OR diarrhoea OR gastroenteritis ) AND TITLE-ABS-KEY ( virus OR viral OR rotavirus OR norovirus OR adenovirus OR astrovirus OR sapovirus ) AND TITLE-ABS-KEY ( Ghana )  TITLE-ABS-KEY ( diarrhea OR diarrhoea OR gastroenteritis ) AND TITLE-ABS-KEY ( rotavirus OR norovirus OR adenovirus OR astrovirus ) AND TITLE-ABS-KEY ( epidemiology OR burden OR prevalence OR incidence OR surveillance OR control OR intervention OR sanitation OR "water supply" ) AND TITLE-ABS-KEY ( Ghana ) |
| **ScienceDirect (435)** | ("diarrhea" OR "gastroenteritis") AND (rotavirus OR norovirus OR adenovirus OR astrovirus OR sapovirus) AND Ghana |

**Study Characteristics**

| Author, Year of publication | Year of Sample  Collection | Location | Sample size | Age Range |
| --- | --- | --- | --- | --- |
| Cohen et al, 2022  (1) | 2017-2018 | KBTH - Accra | 435 | <5 years |
| Lartey et al, 2018  (2) | 2009-2016 |  | 1363 | <5 years |
| Armah et al, 2016  (3) | 2009-2014 | Accra, Kumasi  Ho  Navrongo | 3929 | <5 years |
| Mizutani et al, 2021.  (4) | 2017-2018 | Accra | 80 | ≤45 years |
| Enweronu-Laryea et al, 2014.  (5) | 2006-2011 | Accra | 3044 | <5 years |
| Lartey et al, 2020.  (6) | 2008-2017 | Accra, Kumasi  Ho  Kassena-Nankana  Navrongo  Paga | 1337 | <5 years |
| Damanka et al, 2016.  (7) | 2004-2005 | Accra | 206 | <5 years |
| Enweronu-Laryea et al, 2018.  (8) | 2009-2016 | Accra, Kumasi | 4066 | <5 years |
| Asmah et al, 2001.  (9) | 1998 | Kassena-Nankana | 50 | <2 years |
| Enweronu-Laryea et al, 2014.  (10) | 2008-2014 | Accra | 3160 | <5 years |
| Enweronu-Laryea et al, 2011.  (11) | 2008-2009 | Accra | 1077 | <5 years |
| Krumkamp et al, 2015.  (12) | 2007-2008 | Asante-Akim North | 548 | <13 years |
| Bawa et al, 2023.  (13) | 2020- 2021 | Kassena-Nankana | 263 | <5 years |
| Binka et al, 2011.  (14) | 2009 | Accra | 143 | <5 years |
| Ofosu-Appiah et al, 2025.  (15) | 2019 | Accra | 228 | ≤75 years |
| Armah et al, 2003.  (16) | 1997-2002 | Kassena-Nankana | 2,205 | <2 years |
| Reither et al, 2007.  (17) | 2005-2006 | Tamale | 243 | ≤12 years |
| Ashie et al, 2017.  (18) | 2012-2014 | Kumasi | 240 | <5 years |
| Akuffo et al, 2017.  (19) | 2010- 2012 | Accra  Tamale | 147 | 1 month – 95years |
| Operario et al, 2017.  (20) | 2013-2014 | Ghana | 730 | <5 years |
| Heinemann et al, 2021.  (21) | 2014-2015 | Pramso- Kumasi | 107 | ≤1 years |
| Silva et al, 2008.  (22) | 2005-2006 | Tamale | 367 | ≤1 years |
| Armah et al, 2010.  (23) | 2003-2004 | Navrongo | 289 | <5 years |
| Letsa et al, 2019.  (24) | 2014-2016 | Ashaiman-Accra | 369 | <5 years |
| Enweronu-Laryea et al, 2013.  (25) | 2007-2011 | Accra | 2277 | <5 years |
| Armah et al, 2001.  (26) | 1999 | Navrongo | 67 | <2 years |
| Armah et al, 2006.  (27) | 2004 | Navrongo  Paga  Kassena | 82 | <2 years |
| Armah et al, 2010.  (28) | 1996-2000 | Ghana | 2002 | <2 years |
| Navrongo Research Group, 2003  (29) | 1998-2000 | Kassena-Nankana  Paga  Navrongo | 1717 | <2 years |
| Hori et al, 1996.  (30) | 1992 | Accra | 225 | <5 years |

**Aetiology of Viral Diarrhoea, prevalence and diagnostic methods**

| Author, Year of publication | Aetiology of  viral diarrhoea | Prevalence (%) | Diagnostic method |
| --- | --- | --- | --- |
| Cohen et al, 2022  (1) | Rotavirus  Norovirus  Adenovirus  Sapovirus  Astrovirus | 32.1%  5.8%  5.1%  2.6%  0.9% | Molecular (qPCR) |
| Lartey et al, 2018  (2) | Rotavirus | Pre-rotavirus  vaccination- 60.4%  Post-rotavirus vaccination- 39.6% | Molecular (RT-PCR) |
| Armah et al, 2016  (3) | Rotavirus | Average- 42%  Pre-rotavirus vaccination- 48%  Post-rotavirus vaccination- 28% | Immunologic (EIA) |
| Mizutani et al, 2021.  (4) | Rotavirus  Norovirus | 22.5%  48.7% | Immunologic (EIA)  Molecular (RT-PCR) |
| Enweronu-Laryea et al, 2014.  (5) | Rotavirus | 48.9% | Immunologic (EIA)  Molecular (RT-PCR) |
| Lartey et al, 2020.  (6) | Norovirus | Average- 36.2%  Pre-rotavirus vaccination- 23%  Post-rotavirus vaccination- 48.3% | Molecular (RT-PCR) |
| Damanka et al, 2016.  (7) | Rotavirus | 34% | Immunologic (EIA) Molecular (RT-PCR) |
| Enweronu-Laryea et al, 2018.  (8) | Rotavirus: | Pre-rotavirus vaccination- 50%  Post-rotavirus vaccination- 29% | Immunologic (EIA) |
| Asmah et al, 2001.  (9) |  |  | Molecular (RT-PCR) |
| Enweronu-Laryea et al, 2014.  (10) | Rotavirus | Pre-rotavirus vaccination - 49.7%  Post-rotavirus vaccination 27.8% | Immunologic (EIA) |
| Enweronu-Laryea et al, 2011.  (11) | Rotavirus | 49% | Immunologic (EIA)  Molecular (RT-PCR) |
| Krumkamp et al, 2015.  (12) | Rotavirus  Norovirus | 16.6%  16.2% | Immunologic (ELISA)  Molecular (PCR) |
| Bawa et al, 2023.  (13) | Rotavirus | 14.8% | Semi-nested PCR |
| Binka et al, 2011.  (14) | Rotavirus | 58% | Immunologic (EIA)  Molecular (RT-PCR) |
| Ofosu-Appiah et al, 2025.  (15) | Rotavirus  Norovirus  Adenovirus | 23.7%  11%  5.3% | Immunologic (Antigen Assays)  Molecular (PCR) |
| Armah et al, 2003.  (16) | Rotavirus | 40.5% | Immunologic (EIA)  Molecular (RT-PCR) |
| Reither et al, 2007.  (17) | Rotavirus  Adenovirus  Norovirus  Astrovirus | 54.7%  27.6%  9.5%  4.5% | Molecular (RT-PCR) |
| Ashie et al, 2017.  (18) | Rotavirus  Adenovirus | 26.2%  10.4% | Immunologic (Antigen Assays) |
| Akuffo et al, 2017.  (19) | Rotavirus  Astrovirus  Norovirus  Adenovirus | 7.9%  7.5%  6.8%  5.4% | Immunologic (EIA) |
| Operario et al, 2017.  (20) | Rotavirus | 31% | Immunologic (EIA) |
| Heinemann et al, 2021.  (21) | Rotavirus  Astrovirus  Norovirus  Adenovirus  Sapovirus | 11%  16%  13%  15%  8% | Molecular (PCR) |
| Silva et al, 2008.  (22) | Rotavirus  Astrovirus  Norovirus  Adenovirus | 38.7%  3.3%  7.4%  19.9% | Molecular (RT-PCR) |
| Armah et al, 2010.  (23) | Rotavirus | 39.8% | Immunologic (EIA)  Molecular (RT-PCR) |
| Letsa et al, 2019.  (24) | Rotavirus | 39% | Immunologic (EIA)  Molecular (RT-PCR) |
| Enweronu-Laryea et al, 2013. (25) | Rotavirus | 48.2% | Immunologic (EIA)  Molecular (RT-PCR) |
| Armah et al, 2001.  (26) | Rotavirus | NA | Immunologic (EIA) |
| Armah et al, 2006.  (27) | Norovirus | 15.9% | Molecular (RT-PCR) |
| Armah et al, 2010.  (28) | Rotavirus | 15.9% | Immunologic (EIA) |
| Navrongo Research Group, 2003  (29) | Rotavirus | 39% | Immunologic (EIA)  Molecular (RT-PCR) |
| Hori et al, 1996.  (30) | Rotavirus | 15.1% | Microscopy (Electron microscopy)  Immunologic (EIA) |

**Note: PCR** – Polymerase Chain Reaction, **RT‑PCR** – Reverse Transcription Polymerase Chain Reaction, **qPCR** – Quantitative Polymerase Chain Reaction, **Semi‑nested PCR –** Semi‑nested Polymerase Chain Reaction, **EIA** – Enzyme Immunoassay, **ELIS**A – Enzyme‑Linked Immunosorbent Assay, **NA** – Not available.
**Pre-rotavirus vaccination period** – Before May 2012, **Post-rotavirus vaccination period** – After May 2012.

REFERENCES

1.	Cohen AL, Platts-Mills JA, Nakamura T, Operario DJ, Antoni S, Mwenda JM, et al. Aetiology and incidence of diarrhoea requiring hospitalisation in children under 5 years of age in 28 low-income and middle-income countries: findings from the Global Pediatric Diarrhea Surveillance network. BMJ Glob Health. 2022 Sep 5;7(9):e009548. doi:10.1136/BMJGH-2022-009548 PubMed PMID: 36660904.

2.	Lartey BL, Damanka S, Dennis FE, Enweronu-Laryea CC, Addo-Yobo E, Ansong D, et al. Rotavirus strain distribution in Ghana pre- and post- rotavirus vaccine introduction. Vaccine. 2018 Nov 12;36(47):7238–42. doi:10.1016/J.VACCINE.2018.01.010 PubMed PMID: 29371014.

3.	Armah G, Pringle K, Enweronu-Laryea CC, Ansong D, Mwenda JM, Diamenu SK, et al. Impact and Effectiveness of Monovalent Rotavirus Vaccine Against Severe Rotavirus Diarrhea in Ghana. Clinical Infectious Diseases. 2016 May 1;62(suppl_2):S200–7. doi:10.1093/CID/CIW014 PubMed PMID: 27059357.

4.	Mizutani T, Aboagye SY, Ishizaka A, Afum T, Mensah GI, Asante-Poku A, et al. Gut microbiota signature of pathogen-dependent dysbiosis in viral gastroenteritis. Scientific Reports 2021 11:1. 2021 Jul 6;11(1):13945-. doi:10.1038/s41598-021-93345-y PubMed PMID: 34230563.

5.	Enweronu-Laryea CC, Sagoe KWC, Mwenda JM, Armah GE. Severe acute rotavirus gastroenteritis in children less than 5 years in southern Ghana: 2006-2011. Pediatr Infect Dis J. 2014;33 Suppl 1(SUPPL. 1). doi:10.1097/INF.0000000000000045 PubMed PMID: 24343622.

6.	Lartey BL, Quaye O, Damanka SA, Agbemabiese CA, Armachie J, Dennis FE, et al. Understanding Pediatric Norovirus Epidemiology: A Decade of Study among Ghanaian Children. Viruses. 2020 Nov 1;12(11):1321. doi:10.3390/V12111321 PubMed PMID: 33217894.

7.	Damanka S, Adiku TK, Armah GE, Rodrigues O, Donkor ES, Nortey D, et al. Rotavirus Infection in Children with Diarrhea at Korle-Bu Teaching Hospital, Ghana. Jpn J Infect Dis. 2016;69(4):331–4. doi:10.7883/YOKEN.JJID.2014.407 PubMed PMID: 26370427.

8.	Enweronu-Laryea CC, Armah G, Sagoe KW, Ansong D, Addo-Yobo E, Diamenu SK, et al. Sustained impact of rotavirus vaccine introduction on rotavirus gastroenteritis hospitalizations in children <5 years of age, Ghana, 2009–2016. Vaccine. 2018 Nov 12;36(47):7131–4. doi:10.1016/J.VACCINE.2018.02.058 PubMed PMID: 29752020.

9.	Asmah RH, Green J, Armah GE, Gallimore CI, Gray JJ, Iturriza-Gómara M, et al. Rotavirus G and P genotypes in rural Ghana. J Clin Microbiol. 2001;39(5):1981–4. doi:10.1128/JCM.39.5.1981-1984.2001/ASSET/A488C642-AB69-4F6B-8678-C8B45C5B6900/ASSETS/GRAPHIC/JM0511499001.JPEG PubMed PMID: 11326029.

10.	Enweronu-Laryea CC, Boamah I, Sifah E, Diamenu SK, Armah G. Decline in severe diarrhea hospitalizations after the introduction of rotavirus vaccination in Ghana: A prevalence study. BMC Infect Dis. 2014 Aug 14;14(1):1–6. doi:10.1186/1471-2334-14-431/FIGURES/3 PubMed PMID: 25100574.

11.	Enweronu-Laryea CC, Sagoe KWC, Glover-Addy H, Asmah RH, Mingle JA, Armah GE. Prevalence of severe acute rotavirus gastroenteritis and intussusceptions in Ghanaian children under 5 years of age. The Journal of Infection in Developing Countries. 2011 Nov 30;6(02):148–55. doi:10.3855/jidc.1667 PubMed PMID: 22337844.

12.	Krumkamp R, Sarpong N, Schwarz NG, Adelkofer J, Loag W, Eibach D, et al. Gastrointestinal Infections and Diarrheal Disease in Ghanaian Infants and Children: An Outpatient Case-Control Study. PLoS Negl Trop Dis. 2015 Mar 4;9(3):e0003568. doi:10.1371/JOURNAL.PNTD.0003568 PubMed PMID: 25738935.

13.	Bawa FK, Mutocheluh M, Dassah SD, Ansah P, Oduro AR. Genetic diversity of rotavirus infection among young children with diarrhoea in the Kassena-Nankana Districts of Northern Ghana: a seasonal cross-sectional survey. PAMJ 2023; 44:148. 2023 Mar 28;44(148):148. doi:10.11604/PAMJ.2023.44.148.36783 PubMed PMID: 37396694.

14.	Binka EB, Vermund SH, Armah GE. Rotavirus diarrhea among children less than 5 years of age in urban Ghana. Pediatr Infect Dis J. 2011;30(8):716–8. doi:10.1097/INF.0B013E318223BD85 PubMed PMID: 21646938.

15.	Ofosu-Appiah LH, Negoro M, Amexo JX, Amelor DK, Tonto PB, Laryea DO, et al. Clinical Impact and Genetic Analysis of Enteric Viruses Associated With Acute Gastroenteritis in Greater Accra, Ghana: A Comprehensive Study of Five Viruses. J Med Virol. 2025 Feb 1;97(2):e70216. doi:10.1002/JMV.70216 PubMed PMID: 39935201.

16.	Armah GE, Steele AD, Binka FN, Esona MD, Asmah RH, Anto F, et al. Changing patterns of rotavirus genotypes in Ghana: Emergence of human rotavirus G9 as a major cause of diarrhea in children. J Clin Microbiol. 2003 Jun 1;41(6):2317–22. doi:10.1128/JCM.41.6.2317-2322.2003/ASSET/74849D2B-F81B-4655-8C1A-9F319ACF9530/ASSETS/GRAPHIC/JM0631143002.JPEG PubMed PMID: 12791843.

17.	Reither K, Ignatius R, Weitzel T, Seidu-Korkor A, Anyidoho L, Saad E, et al. Acute childhood diarrhoea in northern Ghana: epidemiological, clinical and microbiological characteristics. BMC Infect Dis. 2007 Sep 6;7(1):104. doi:10.1186/1471-2334-7-104/TABLES/2 PubMed PMID: 17822541.

18.	Ashie GK, Mutocheluh M, Owusu M, Kwofie TB, Akonor S, Narkwa PW, et al. Microbial pathogens associated with acute childhood diarrhoea in Kumasi, Ghana. BMC Res Notes. 2017 Jul 11;10(1):1–7. doi:10.1186/S13104-017-2578-9/FIGURES/1 PubMed PMID: 28693616.

19.	Akuffo R, Armah G, Clemens M, Kronmann KC, Jones AH, Agbenohevi P, et al. Prevalence of enteric infections among hospitalized patients in two referral hospitals in Ghana. BMC Res Notes. 2017 Jul 17;10(1). doi:10.1186/S13104-017-2621-X PubMed PMID: 28716138.

20.	Operario DJ, Platts-Mills JA, Nadan S, Page N, Seheri M, Mphahlele J, et al. Etiology of Severe Acute Watery Diarrhea in Children in the Global Rotavirus Surveillance Network Using Quantitative Polymerase Chain Reaction. J Infect Dis. 2017 Jul 15;216(2):220–7. doi:10.1093/INFDIS/JIX294 PubMed PMID: 28838152.

21.	Heinemann M, Strauchs C, Lütgehetmann M, Aepfelbacher M, Klupp EM, Owusu-Dabo E, et al. Polymicrobial enteric infections in African infants with diarrhoea—results from a longitudinal prospective case–control study. Clinical Microbiology and Infection. 2021 Dec 1;27(12):1792–8. doi:10.1016/j.cmi.2021.03.020 PubMed PMID: 33813114.

22.	Silva PA, Stark K, Mockenhaupt FP, Reither K, Weitzel T, Ignatius R, et al. Molecular characterization of enteric viral agents from children in northern region of Ghana. J Med Virol. 2008 Oct 1;80(10):1790–8. doi:10.1002/JMV.21231 PubMed PMID: 18712819.

23.	Armah GE, Hoshino Y, Santos N, Binka F, Damanka S, Adjei R, et al. The Global Spread of Rotavirus G10 Strains: Detection in Ghanaian Children Hospitalized with Diarrhea. J Infect Dis. 2010 Sep 1;202(Supplement_1):S231–8. doi:10.1086/653572 PubMed PMID: 20684709.

24.	Letsa V, Damanka S, Dennis F, Lartey B, Armah GE, Betrapally N, et al. Distribution of rotavirus genotypes in the postvaccine introduction era in Ashaiman, Greater Accra Region, Ghana, 2014-2016. J Med Virol. 2019 Nov 1;91(11):2025–8. doi:10.1002/JMV.25542 PubMed PMID: 31286526.

25.	Enweronu-Laryea CC, Sagoe KW, Damanka S, Lartey B, Armah GE. Rotavirus genotypes associated with childhood severe acute diarrhoea in southern Ghana: A cross-sectional study. Virol J. 2013 Sep 14;10(1):1–6. doi:10.1186/1743-422X-10-287/FIGURES/2 PubMed PMID: 24034588.

26.	George E Armah, Cara T Pager, Richard H. Asmah, Francis R. Anto, Abraham Rexdford Oduro, Fred Binka, et al. Prevalence of unusual human rotavirus strains in Ghanaian children [Internet]. 2001 [cited 2026 Apr 7]. p. 67–71. Available from: https://onlinelibrary.wiley.com/doi/epdf/10.1002/1096-9071%28200101%2963%3A1%3C67%3A%3AAID-JMV1010%3E3.0.CO%3B2-T PubMed PMID: 11130890.

27.	Armah GE, Gallimore CI, Binka FN, Asmah RH, Green J, Ugoji U, et al. Characterisation of norovirus strains in rural Ghanaian children with acute diarrhoea. J Med Virol. 2006 Nov 1;78(11):1480–5. doi:10.1002/JMV.20722 PubMed PMID: 16998875.

28.	Armah GE, Steele AD, Esona MD, Akran VA, Nimzing L, Pennap G. Diversity of rotavirus strains circulating in west Africa from 1996 to 2000. J Infect Dis. 2010 Sep 1;202 Suppl(SUPPL. 1). doi:10.1086/653571 PubMed PMID: 20684720.

29.	Navrongo T, Research Group R. Incidence and risk factors of paediatric rotavirus diarrhoea in northern Ghana. Tropical Medicine & International Health. 2003 Sep 1;8(9):840–6. doi:10.1046/J.1365-3156.2003.01097.X PubMed PMID: 12950670.

30.	Hori H, Akpedonu P, Armah G, Aryeetey M, Yartey J, Kamiya H, et al. Enteric pathogens in severe forms of acute gastroenteritis in Ghanaian children. Pediatrics International. 1996 Dec 1;38(6):672–6. doi:10.1111/J.1442-200X.1996.TB03729.X;PAGE:STRING:ARTICLE/CHAPTER PubMed PMID: 9002307.
